# Galectin-3 in Patients with Atrial Fibrillation Undergoing Radiofrequency Catheter Ablation

**DOI:** 10.1371/journal.pone.0123574

**Published:** 2015-04-15

**Authors:** Jelena Kornej, Josephin Schmidl, Laura Ueberham, Silke John, Sait Daneschnejad, Borislav Dinov, Gerhard Hindricks, Volker Adams, Daniela Husser, Andreas Bollmann

**Affiliations:** 1 Department of Electrophysiology, Heart Center, Leipzig, Germany; 2 Department of Cardiology, Heart Center, Leipzig, Germany; University at Buffalo, UNITED STATES

## Abstract

**Background:**

Galectin-3 (Gal-3) is an emerging biomarker in heart failure that is involved in fibrosis and inflammation. However, its potential value as a prognostic marker in atrial fibrillation (AF) is unknown. The aim of this study was to assess the impact of AF catheter ablation on Gal-3 and evaluate its prognostic impact for predicting rhythm outcome after catheter ablation.

**Methods:**

Gal-3 was measured at baseline and after 6 months using specific ELISA. AF recurrences were defined as any atrial arrhythmia lasting longer than 30 sec within 6 months after ablation.

**Results:**

In 105 AF patients (65% males, age 62±9 years, 52% paroxysmal AF) undergoing catheter ablation, Gal-3 was measured at baseline and after 6 months and compared with an AF-free control cohort (n=14, 50 % males, age 58±11 years). Gal-3 was higher in AF patients compared with AF-free controls (7.8±2.9 vs. 5.8±1.8, ng/mL, p=0.013). However, on multivariable analysis, BMI (p=0.007) but not AF (p=0.068) was associated with Gal-3. In the AF cohort, on univariable analysis higher Gal-3 levels were associated with female gender (p=0.028), higher BMI (p=0.005) and both CHADS2 (p=0.008) and CHA2DS2-VASC (p=0.016) scores, however, on multivariable analysis only BMI remained significantly associated with baseline Gal-3 (p=0.016). Gal-3 was similar 6 months after AF catheter ablation and was not associated with sinus rhythm maintenance.

**Conclusions:**

Although galectin-3 levels are higher in AF patients, this is driven by cardiometabolic co-morbidities and not heart rhythm. Gal-3 is not useful for predicting rhythm outcome of catheter ablation.

## Introduction

Several blood biomarkers have been identified which are helpful in supporting atrial fibrillation (AF) diagnosis, prognosis and outcomes. Since catheter ablation has become the cornerstone of non-pharmacological AF treatment the interest for blood biomarkers reflecting and predicting outcomes after this treatment strategy has rapidly grown [1].

Natriuretic peptides have previously been established for a variety of cardiovascular diseases as important markers of increased mortality and morbidity, e.g. in acute coronary syndromes, stable coronary disease or congestive heart failure. Recently, Hijazi et al. [2] demonstrated that elevation of NT-proBNP was independently related to increased risks of stroke and mortality in AF patients. Furthermore, baseline NT-proBNP level correlated with AF burden in patients with lone AF and independently predicted AF recurrences after catheter ablation [3]. Some studies demonstrated reduction of transcardiac BNP levels after AF catheter ablation emphasizing the significance of the left atrium as initial origin for BNP production in AF [4].

Because of its involvement in cardiac fibrosis, inflammation and remodelling processes, galectin-3 (Gal-3) is one of the emerging biomarkers in cardiac diseases [5]. Together with NT-pro-BNP, Gal-3 demonstrated better prognostic value for mortality in general population and in patients with heart failure [6]. AF is associated with structural remodeling based on pro-inflammatory and pro-fibrotic changes in atrial tissue, thus suggesting an association between Gal-3 and AF. A recently published analysis of more than 3,000 participants of the Framingham Offspring cohort found that higher circulating Gal-3 concentrations were associated with increased risk of developing AF [7].

However, there are no systematic analyses of this biomarker in AF available and a possible association with rhythm outcomes after catheter ablation has not been investigated. Consequently, this study, for the first time, (i) compared Gal-3 in AF patients with a AF-free cohort, (ii) assessed trans-cardiac and trans-pulmonary gradients, (iii) assessed the impact of AF catheter ablation, and (iv) evaluated its potential prognostic impact for predicting rhythm outcome.

## Methods

### Study population

We recruited 105 patients with symptomatic AF who underwent radiofrequency catheter ablation at Heart Center Leipzig, Germany and fourteen AF-free controls matched for age, gender and heart disease. The study was approved by the local ethics committee (Medical Faculty, Leipzig University) and patients provided written informed consent for participation. Paroxysmal and persistent AF was defined according to current guidelines [8]. Paroxysmal AF was defined as self-terminating within 7 days after onset documented by previous routine electrocardiograms (ECG) or Holter ECG. Persistent AF was defined as any AF episode either lasting longer than 7 days or requiring drug or direct current cardioversion for termination. AF-free controls without AF-related symptoms were recruited from the outpatient clinic and freedom from AF was verified by previous ECG reports.

In all AF patients, transthoracic and transesophageal echocardiography was performed prior to ablation. All class I or III antiarrhythmic medications with the exception of amiodarone were discontinued at least 5 half-lives before the procedure. Estimated glomerular filtration rate (eGFR) was calculated by using the Cockroft-Gault equation: (140—age) x weight (kg) x (0.85 if female) / 72 x serum creatinine (mg/dl).

### Radiofrequency catheter ablation

Patients presenting with AF at the beginning of the procedure were electrically cardioverted and ablation was performed during sinus rhythm (i.e. AF termination with ablation was not attempted). Pulmonary vein (PV) isolation was performed by sequential application of radiofrequency energy at the antrum of the pulmonary veins. End-point was isolation of the PV with proof of both exit and entrance block. After the isolation of the pulmonary veins, electoanatomical voltage maps of the LA body in sinus rhythm were created in each patient using ablation catheter as a roving catheter. Potentials with amplitudes over 0.5 mV were defined as normal, and potentials under 0.2 mV as low-voltage. According to the underlying substrate and induced left atrial macro reentry tachycardias (LAMRT) additional lines transecting the scar areas to connect with healthy tissue or anatomical obstacles were ablated.

After ablation, class I and III antiarrhythmic drugs were not reinitiated. Proton pump inhibitors were added for 4 weeks. According to the current guidelines [8], oral anticoagulation was prescribed for 3–6 months after catheter ablation and depending on risk stratification of stroke using the CHA_2_DS_2_-VASc score thereafter.

### Follow-up

All patients were followed in the outpatient clinic for 6 months after catheter ablation. During this follow-up period, 7-days Holter ECG recordings were performed (immediately, 3 and 6 months after the ablation). Additional ECGs and Holter ECG recordings were obtained when patients’ symptoms were suggestive of AF. Early AF recurrences (ERAF) were defined as any atrial arrhythmia lasting >30 seconds within first week after catheter ablation, while late AF recurrences (LRAF) were any episodes between 3 and 6 months after ablation.

### Blood samples

Blood samples were obtained before and 6 months after catheter ablation from peripheral vein. In a sub-group of 10 patients, additional blood samples were taken from cardiac circulation (coronary sinus and left atrium) before ablation.

Platelet-poor plasma fractions were obtained by centrifugation at 20°C for 10 min at 3500 × g, and plasma were stored at -80°C for subsequent analysis. Galectin-3 plasma levels were quantified using commercially available specific enzyme-linked immunoabsorbent assays (ELISA) according to manufacturer protocol (Hölzel Galectin-3 (human) ELISA HZ-4858, Cologne, Germany). Intra-assay coefficient of variation for ELISA assay was <5%, and inter-assay variance was <10%. Results were compared with standard curves and the lower detection limit was 0.12 ng/ml.

### Statistical analysis

Data are presented as mean and standard deviation (SD) or median [interquartile range] for continuous variables and as proportions for categorical variables. Comparison of continuous variables was performed using the unpaired Student’s t-test and of categorical variables using the Pearson chi-square test. To investigate the relationship between Gal-3 plasma levels and clinical variables, linear regression was performed with Gal-3 plasma concentration as dependent variable (univariable analysis, UV). Logistic regression analysis was used to identify factors associated with AF recurrences after catheter ablation. Multivariable analyses (MV), which included variables with a p-value <0.1 found on univariable analysis, were performed to identify independent associations between clinical variables and baseline Gal-3 levels as well as rhythm outcomes. Comparison of biomarker plasma levels in the cardiac and peripheral circulation was performed using the Student’s paired t-test to identify a potential transcardiac gradient, i.e. comparison of left atrium (LA) and coronary sinus (CS) levels, and to identify a potential transpulmonary gradient, i.e. comparison of LA and peripheral vein (peV) levels.

A p-value <0.05 was considered as statistically significant. Statistical analyses were performed with SPSS statistical software version 17.

## Results

### Gal-3 plasma levels in AF

**Fig 1:**
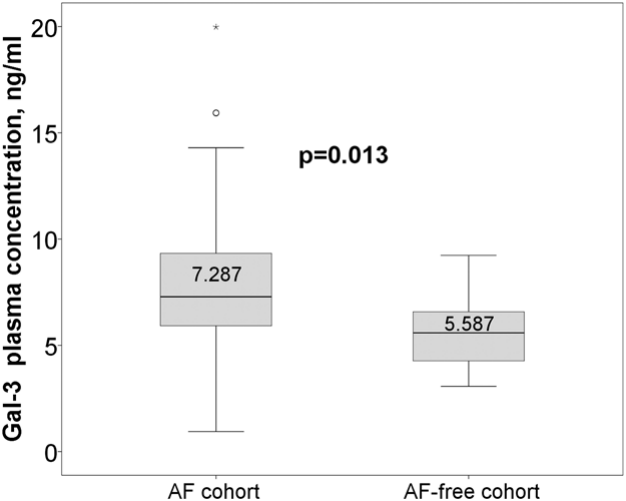
Gal-3 plasma levels in patients with AF and AF-free cohort.

Baseline characteristics of the study population are summarized in Table 1. There were no significant differences between AF and AF-free patients. AF patients had significantly higher Gal-3 levels than AF-free controls (7.8±2.9 vs. 5.8±1.8, ng/mL, p = 0.013; Fig 1). However, on multivariable analysis, BMI (Beta = .250, p = 0.007) but not AF (Beta = .166, p = 0.068) was associated with Gal-3 levels (Table 2).

**Table 1:** Baseline characteristics of the study population.

	AF patients (n = 105)	AF-free controls (n = 14)	*P*-value
Age, years	62± 9	58 ± 11	0.105
Males (%)	65	50	0.282
Paroxysmal AF (%)	51	-	-
Lone AF (%)	71	-	-
AF history, months	60 (2–540)	-	-
CHADS_2_-Score	1 (0–4)	-	-
CHA_2_DS_2_-VASc-Score	2 (0–7)	-	-
BMI, kg/m^2^	29 ± 5	26 ± 4	0.074
LAD, mm	42 ± 6	39 ± 7	0.115
LVEF (%)	60 ± 8	59 ± 11	0.787
eGFR, ml/min	97 ± 30	100 ± 31	0.759

**Abbreviations:** BMI—body mass index; LAD—left atrial diameter; LVEF—left ventricular ejection fraction; eGFR—estimated glomerular filtration rate

Data are presented as median (interquartile range) or mean value ± SD.

**Table 2:** Clinical parameters associated with baseline Gal-3 plasma levels in study population.

Variables	UV		MV	
	Beta	*P*-value	Beta	*P*-value
Age	.215	0.019	.145	0.136
Females	.180	0.051	.120	0.212
AF	.227	0.013	.166	0.068
BMI	.295	0.001	.250	0.007
Hypertension	.155	0.092	.051	0.578
LAD	.081	0.381		
LVEF	.025	0.785		
eGFR	-.097	0.303		

**Abbreviations**: as in Table 1; UV—univariable model, MV—multivariable model.

In the AF cohort, on univariable analysis higher Gal-3 levels were associated with female gender (Beta = .165, p = 0.028), higher BMI (Beta = .203, p = 0.005) and both CHADS_2_ (Beta = .174, p = 0.008) and CHA_2_DS_2_-VASC (Beta = .185, p = 0.016) scores. However, on multivariable analysis only BMI remained significantly associated with baseline Gal-3 levels (Beta = .235, p = 0.016) while female gender and both risk scores showed a trend for association (Table 3).

**Table 3:** Clinical parameters associated with baseline Gal-3 plasma levels in AF patients.

Variables	UV		MV*	Model 1	MV	Model 2
	Beta	*P*-value	Beta	*P*-value	Beta	*P*-value
Age	.167	0.088				
Females	.215	0.028	.165	0.081		
Persistent AF	.076	0.438				
BMI	.274	0.005	.203	0.040	.235	0.016
eGFR	-.080	0.422				
LAD	.045	0.652				
LVEF	.021	0.831				
CHADS_2_	.259	0.008	.174	0.077		
CHA_2_DS_2_-VASc	.234	0.016			.185	0.055

**Abbreviations**: as in Table 1 and 2.

*We performed multivariable analysis separately for CHADS_2_ and CHA_2_DS_2_-VASc score (MV model 1 for the CHADS_2_, MV model 2 for the CHA_2_DS_2_-VASc score).

### Gal-3 levels in cardiac and peripheral circulation

**Fig 2:**
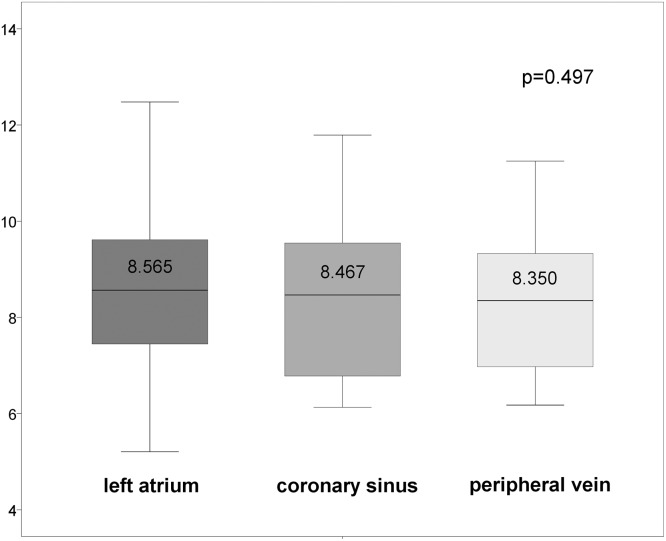
Gal-3 plasma levels in cardiac and peripheral circulation.

In 10 patients the levels between Gal-3 plasma concentrations in the left atrium (LA), coronary sinus (CS) were comparable with Gal-3 levels in peripheral vein (peV). Therefore, no trans-cardiac and trans-pulmonary gradients could be found (Fig 2).

### Gal-3 as predictor for rhythm outcomes after catheter ablation

Comparison of baseline characteristics and Gal-3 plasma levels in patients with and without AF recurrences is represented in Table 4. Patients with ERAF had more often persistent AF than patients without recurrence (p = 0.04). There were no significant differences in clinical characteristics between patients with and without LRAF.

**Table 4:** Clinical characteristics and Gal-3 plasma levels at baseline and 6 months after catheter ablation in patients with and without AF recurrences.

	ERAF*			LRAF#		
	Yes (n = 40)	No (n = 55)	*P*-value	Yes (n = 36)	No (n = 56)	*P*-value
Age, years	61 ± 9	63 ± 10	0.382	63 ± 9	61 ± 10	0.429
Males (%)	68	66	0.835	69	64	0.609
Paroxysmal AF (%)	40	62	0.035	42	61	0.074
Lone AF (%)	68	76	0.339	61	80	0.043
AF history, months (median, IQR)	60 (2–480)	60 (3–540)	0.888	36 (2–216)	60 (3–540)	0.181
CHADS_2_ (median, IQR)	1 (0–4)	1 (0–4)	0.919	1 (0–4)	1 (0–3)	0.016
CHA_2_DS_2_-VASc (median, IQR)	2 (0–7)	2 (0–6)	0.541	2 (0–7)	2 (0–6)	0.207
BMI	29 ± 5	29 ± 5	0.687	30 ± 6	28 ± 5	0.156
LAD, mm	43 ± 7	42 ± 6	0.395	43 ± 7	41 ± 6	0.248
LVEF, %	60 ± 10	60 ± 7	0.708	60 ± 11	60 ± 7	0.901
eGFR, ml/min	100 ± 37	94 ± 26	0.343	95 ± 36	98 ± 28	0.671
Baseline Gal-3, ng/ml	7.47 ± 2.40	8.01 ± 3.37	0.382	7.08 ± 2.67	8.09 ± 3.15	0.115
FU Gal-3, ng/ml	-	-	-	7.09 ± 2.75	8.11 ± 3.13	0.111

**Abbreviations**: as in the Table 1, ERAF—early AF recurrences, LRAF—late AF recurrences, FU—6 months follow-up.

*Data available in 95 patients

^#^Data available in 92 patients

**Fig 3:**
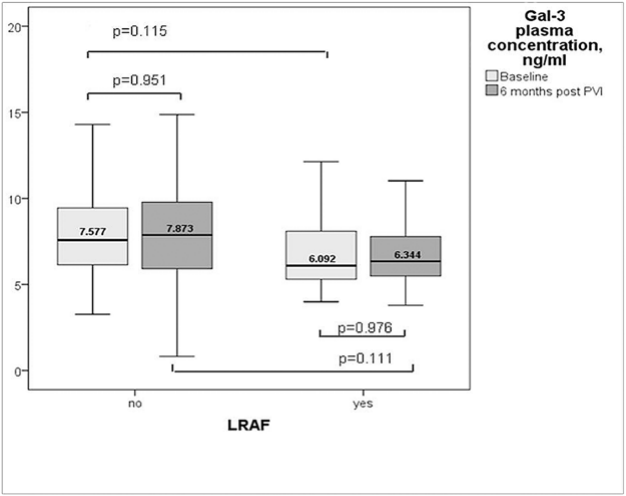
Gal-3 plasma levels in AF patients with and without AF recurrences.

There were no significant differences in Gal-3 plasma levels in patients with and without ERAF (n = 0.382). Similar, Gal-3 levels at baseline and at 6 months were comparable in patients with and without LRAF (Fig 3). On multivariable analysis, only persistent AF (OR 2.84, 95%CI 1.12–7.19, p = 0.003) and lone AF (OR 0.29, 95%CI 0.11–0.80, p = 0.02) remained associated with AF recurrence.

## Discussion

### Main findings

To the best of our knowledge, this study is the first to investigate the potential role of Gal-3 in AF and their response to catheter ablation. Several findings are of special importance: (i) Gal-3 levels are higher in AF patients than in AF-free controls, however, this seems to be mediated by higher BMI than by heart rhythm; (ii) there are no differences in Gal-3 trans-cardiac and trans-pulmonary levels; (iii) Gal-3 was not associated with early and late rhythm outcomes after AF catheter ablation.

### Galectin-3 in AF

Gal-3 had been shown as emerging biomarker for the prediction of mortality in patients with heart failure [6]. Because of inflammatory and pro-fibrotic processes leading to the structural remodeling in both diseases, heart failure is associated with AF and was even included into thromboembolic risk stratification scores. However, the role of Gal-3 in AF is understudied. Recently, Ho et al [7] demonstrated an association between higher circulating Gal-3 concentrations and increased risk of developing AF, however, this study failed to demonstrate, that Gal-3 levels could be used to predict AF after adjustment for traditional clinical risk factors. De Boer et al [9] demonstrated that elderly population and women have higher Gal-3 levels that also was confirmed in our study. In contrast to recently published study by Gurses et al [10] we failed to demonstrate higher Gal-3 levels in patients with persistent AF. Although similarly to previous study we found higher Gal-3 levels in patients with AF than in controls, further analyses revealed the impact of underlying co-morbidity (reflected by the CHADS_2_ and CHA_2_DS_2_-VASc scores) than the rhythm per se. The gender related differences in Gal-3 levels might be explained by hormonal regulation while relation with other AF related co-morbidities (such as diabetes) has recently been investigated [11]. This study also demonstrated higher Gal-3 levels in obese patients that together with diabetes mellitus represent an important part of metabolic syndrome. We confirm these results by showing a significant association between higher BMI and baseline Gal-3 levels in both the entire and the AF cohort.

Several studies have compared biomarker levels in peripheral and cardiac circulation [4, 12]. For instance, inflammation is a known modulating factor in AF arrhythmogenesis and contributes to AF maintenance and perpetuation. Recently, Marcus et al demonstrated significantly lower levels of inflammatory markers in the coronary sinus than in the femoral vein in patients in atrial flutter, while patients in sinus rhythm showed no such difference. Thus, it had been suggested that atrial arrhythmias result in a collection of inflammatory cytokines in the heart, a process that could contribute to adverse remodeling [12].

Interestingly, in the non-failing heart, the source of BNP is believed to be in the atrium and BNP mRNA has been found in the left atrial wall [13]. Recently, it has been demonstrated that patients with paroxysmal AF have significantly higher BNP levels in cardiac circulation than AF-free controls [4]. Furthermore, ablation of the left atrium was associated with an immediate decrease of BNP levels, implicating this as the source. We also found higher Gal-3 levels in AF patients; however, differences in Gal-3 levels between the cardiac (coronary sinus and left atrium) and the peripheral circulation could not be found. A possible explanation could be the relation with systemic inflammation and/or collagen turnover rather than the cardiac source of these markers [14].

### Galectin-3 as predictor for the rhythm outcomes after catheter ablation

Several studies were performed to identify *clinical* predictors for AF recurrences after catheter ablation. Among those persistent AF and enlarged left atrial diameter have reproducibly been shown to associate with AF recurrences [15]. Recently, in a large contemporary AF ablation cohort we found that all three stroke risk stratification scores (i.e. CHADS_2_, CHA_2_DS_2_-VASc and R_2_CHADS_2_) were significantly associated with rhythm outcomes within 12 months after AF catheter ablation [16].

Numerous studies evaluated the impact of inflammatory and pro-fibrotic markers in the prediction of rhythm outcomes after catheter ablation. Inflammatory markers, as a part of oxidative stress, can be related to both early and late AF recurrences after catheter ablation [17]. Recently, we indicated the possible role between inflammatory and auto-immune involvement and AF maintenance and progression, demonstrating an association between HSP70 and anti-HSP70 antibodies with early and late recurrences after catheter ablation [18]. Of interest, circulating HSP70 may act as a pro-fibrotic regulator of the extracellular matrix proteins synthesis through changes in the cytokine TGF-β1 production [19].

BNP (B-type natriuretic peptide) is a neurohormone secreted from the myocytes as response to increased wall tension by volume or pressure overload. It has been shown that baseline NT-proBNP levels are an independent predictor of AF recurrence after catheter ablation [3]. Interestingly, in patients with lone AF BNP levels correlated with AF burden and were also strongly associated with recurrent arrhythmia after ablation [20]. However, despite close relation of BNP and Gal-3 as markers for cardiovascular remodeling, we did not find the association between baseline Gal-3 levels and rhythm outcomes after catheter ablation. A possible explanation could be that both biomarkers might have different levels of sensitivity concerning cardiac rhythm. It has been already demonstrated that Gal-3 had a lower specificity and sensitivity to identify acute heart failure patients compared to NT-proBNP [21]. Furthermore, there are some differences in the pathophysiological mechanisms underlying the secretion of both biomarkers. Whereas BNP act as “loading biomarker”, representing volume or pressure overload in the atria, Gal-3 is associated with turnover of the extracellular matrix and inflammation [22]. Therefore, Gal-3 plasma levels, measured in our study, are more likely reflecting a symbiosis of different cofounding variables (as age, gender, co-morbidities) as the cardiac rhythm per se also after catheter ablation.

### Limitations

This study has several limitations. First, the study cohort included 105 consecutive Caucasian patients with different AF forms. Furthermore, our control group was relatively small and might be not sufficient to demonstrate significant differences in Gal-3 levels in patients with and without AF. Similar, the sub-group with different blood compartments was small and not significant or borderline results found in our population should be interpreted with caution but such sample sizes are usually used in first exploratory studies [23]. The Gal-3 levels found in our study were lower than in the study by Ho et al [7]. This might be partly explained by different assays and very high participant number. However, despite this inconsistence, another studies reported similar or even lower Gal-3 levels [10, 11].

Monitoring of AF recurrence was limited to serial 7-day Holter ECGs which is in line with current guidelines but this strategy may nevertheless have missed asymptomatic AF recurrences. Despite several definitions for early AF recurrences, in current study ERAF was considered as any atrial arrhythmia lasting longer than 30 sec and occurring within first week after ablation. Although the rate of ERAF could be underestimated due to non-inclusion of AF recurrences within the whole 3 months blanking period, from a practical point this seems to be an ideal scenario since alterations in management can be provided quite early.

Finally, this analysis was restricted to the measurement of Gal-3 and did not include other biomarkers.

## Conclusions

Although Gal-3 levels are higher in AF patients, this is driven cardiometabolic co-morbidities and not heart rhythm. Gal-3 is not useful for predicting rhythm outcome of catheter ablation. Further studies are needed to define a potential role of Gal-3 in the AF population.
